# Editorial: Sedation and analgesia challenges in critically ill neonates and children

**DOI:** 10.3389/fped.2022.1003736

**Published:** 2022-08-18

**Authors:** Angela Amigoni, Sinno Simons, Matthijs De Hoog, Saskia N. De Wildt, Oliver Karam

**Affiliations:** ^1^Pediatric Intensive Care Unit, Department of Woman's and Child's Health, University Hospital of Padova, Padova, Italy; ^2^Division of Neonatology, Department of Pediatrics, Erasmus University Medical Center, Rotterdam, Netherlands; ^3^Department of Pediatric Surgery, Erasmus MC—Sophia Children's Hospital, University Medical Center, Rotterdam, Netherlands; ^4^Department of Intensive Care Medicine, Radboud University Medical Center, Nijmegen, Netherlands; ^5^Section of Pediatric Critical Care, Department of Pediatrics, Yale School of Medicine, New Haven, CT, United States

**Keywords:** sedation, analgesia, pediatrics, critical care (ICU), neonates, withdrawal, delirium

Adequate analgesia and sedation are a precondition in the treatment of critically ill neonates and children in different clinical settings. Controlling pain and agitation enables safe mechanical ventilation and invasive procedures, decreases oxygen demand, while it also decreases accidental removal of medical devices. An appropriate sedative and analgesic approach may also promote better medical outcomes and reduce the risk of patients' complications. Both under- and over-treatment are detrimental for patients. Undersedation does not allow to obtain appropriate control of distress, with physiological and physical consequences, and does not promote optimization of mechanical ventilation. Oversedation may cause side effects, may harm the developing central nervous system, delay recovery, cause tolerance, and increase the incidence of withdrawal syndrome and pediatric delirium.

Recently, reference guidelines on analgesia and sedation were published (1, 2). However, despite these recommendations, clinical challenges and difficulties persist because many areas with weak level of evidence are reported and some decisions are left to the clinicians' expertise. Knowledge gaps for specific groups of critically ill pediatrics patients, difficult sedation and analgesia in clinical practice, neuromonitoring during neuromuscular blockade, sedation strategies in various settings, and environment optimizing bundles are all areas that need further investigation to identify the best approach. Moreover, the implementation of protocols of analgesia and sedation and their sustainability are challenging, as demonstrated by Yang et al.

The current literature is very clear on a few aspects. The presence of pain needs to be considered and treated, and the level of sedation should be titrated according to the patient's status and the clinical situation. In addition, the sedation plan should be suitable for the individual patient and flexible (1, 3).

To address pain, many molecules are suggested, depending on the intensity and level of pain. For severe pain opioids are warranted. One of these molecules, hydromorphone, has been extensively reviewed by Rodieux et al. Pharmacokinetics and pharmacodynamics of this drug at younger ages are not well-known. For this reason, current data do not support any advantage of the use of hydromorphone over morphine in children, both in terms of efficacy and safety. Regarding pain treatment in preterm neonates paracetamol/acetaminophen still is an under-evaluated analgesic therapy. In a retrospective monocentric study exploring the use of paracetamol after surgery in extremely low birth weight preterm infants, Cihlarova et al. reported its safety, although the opioid-sparing effect remains to be evaluated.

One of the key concepts of implementing analgesia and sedation in a PICU is to follow a standardized algorithm, assessing and re-assessing pain, distress and/or level of sedation, iatrogenic withdrawal syndrome and delirium. Three papers in this Research Topic discuss assessment tools, paving the way for successful use in clinical practice. Mencía et al. reported the efficacy of implementing an analgo-sedation assessment by clinical scales, which included a multicenter protocol and staff training. The effects of these interventions were measured with a survey, comparing participating and non-participating centers as well as a before-and-after analysis. In the post-intervention phase, an increased use of protocols and monitoring scales were reported. Tapia et al. analyzed the validity and the reliability of the Richmond Agitation-Sedation Scale (RASS) in a multicenter study. They showed an excellent inter-rater reliability and a good correlation between RASS and the COMFORT-B scale. Of note, these results apply to patients with adequate sedation, but also to those who are under- or over-sedated. Finally, Fazio et al. assessed the validity of the Italian translation of the Cornell Assessment of Pediatric Delirium (CAPD) scale. In this single-center study, the authors showed a high intra- and inter-rater agreement. In addition, they showed that the CAPD scale to have a higher accuracy in diagnosing delirium when compared to clinical evaluation by an intensivist.

Delirium, a well-described entity in adult patients, has become a mainstream interest in PICU research these recent years. Many studies evaluated effects of different delirium related approaches to critically ill children, not only to prevent delirium's complication, but also to increase the quality of care and to reduce the sequelae of intensive care treatments (4, 5). The ABCDEF-Bundle include: (A) assessment, prevention and management of pain, (B) spontaneous awakening and breathing trials, (C) choice of analgesia and sedation, (D) assessment, prevention, and management of delirium, (E) early mobility and exercise and (F) family engagement and empowerment. Engel et al. reviewed the literature in the field and proposed modified ABCDEF bundles for children. The authors' message is to implement these strategies in all pediatric intensive care units, advocating for pediatric studies, as today's literature is scarce.

Michel et al. conducted a single-center study to evaluate the impact of delirium bundles, comparing pre- and post-implementation data. They reported a significant reduction of the incidence of delirium, particularly in younger children and in patients after surgery for congenital heart disease.

Finally, this Research Topic addressed two long-term consequences of critical illness, at least in part due to analgesia and sedation. Neurological (including critical illness myopathy and polyneuropathy), cognitive, social, or mental health disease are well-described in adults after critical illness. Post-intensive care syndrome, or PICS, is a term used to define sequelae after intensive care. The acronym is PICS-p when affecting children, and PICS-f when affecting the family or caregivers (6, 7). A survey, conducted by Von Borell et al. in German-speaking PICUs, evaluated the staff (physicians, nurses, and psychotherapists) knowledge of PICS. The authors reported that a minority of respondents believed PICS to be of importance in their daily clinical practice.

Finally, van den Bosch et al. published a study that followed five cohorts of neonates that were exposed to pain, opioids, or anesthetics. The authors showed that while there weren't major cognitive effects eight to 19 years after exposure in early life to neonatal pain, opioid or anesthetic exposure in children, patients with the highest doses (neonates on extracorporeal membrane oxygenation and neonates with prenatal opioid exposure) had worse neuropsychological functioning.

As demonstrated by this Research Topic on *Sedation and analgesia challenges in critically ill neonates and children*, there are multiple knowledge gaps. Further efforts are warranted to establish the best strategy of analgesia and sedation in neonates and children, assessing both efficacy and safety. Protocols, based on a regular re-assessment of pain, agitation, withdrawal symptoms and delirium, are likely to be beneficial and should be implemented, even if their implementation and long-term sustainability is challenging.

## Author contributions

AA wrote the first draft, which was revised and edited by OK. All authors contributed to this editorial final version.

## Conflict of interest

The authors declare that the research was conducted in the absence of any commercial or financial relationships that could be construed as a potential conflict of interest.

## Publisher's note

All claims expressed in this article are solely those of the authors and do not necessarily represent those of their affiliated organizations, or those of the publisher, the editors and the reviewers. Any product that may be evaluated in this article, or claim that may be made by its manufacturer, is not guaranteed or endorsed by the publisher.
